# Supplementary material for: Structural insight into the molecular mechanism of p53-mediated mitochondrial apoptosis
Source: Nat Commun. 2021 Apr 16;12:2280. doi: 10.1038/s41467-021-22655-6 (PMC8052441; doi:10.1038/s41467-021-22655-6)
Supplement: Supplementary file 1 — Supplementary Information [file 41467_2021_22655_MOESM1_ESM.pdf]

# Supplementary Materials for

## Structural Insight into the Molecular Mechanism of p53-mediated Mitochondrial Apoptosis

Hudie Wei[1], Lingzhi Qu[1], Shuyan Dai[1], Yun Li[1], Haolan Wang[1], Yilu Feng[1], Xiaojuan Chen[1], Longying Jiang[1], Ming Guo[1], Jun Li[1], Zhuchu Chen[1], Lin Chen[2], Ye Zhang[1,*], Yongheng Chen[1,3,*]

[1] Department of Oncology, NHC Key Laboratory of Cancer Proteomics, Laboratory of Structural Biology, Xiangya Hospital, Central South University, Changsha, Hunan 410008, China.

[2]Molecular and Computational Biology Program, Department of Biological Sciences and Department of Chemistry, University of Southern California, Los Angeles, California 90089, United States

[3]National Clinical Research Center for Geriatric Disorders, Xiangya Hospital, Central South University, Changsha, Hunan 410008, China.

[*]To whom correspondence should be addressed. Tel: +86 731 84327542; Fax: +86 731 84327542; Email: yonghenc@163.com (Y.C.), yezhang90@csu.edu.cn (Y.Z.)

This PDF file includes:

- Supplementary Tables 1-2
- Supplementary Figures 1-15

**Supplementary Table 1. Data collection and refinement statistics**

| | p53/BCL-xL structure |
|---|---|
| **Data collection** | |
| Wavelength (Å) | 0.97 |
| Space group | P 1 21 1 |
| Cell dimensions | |
| a, b, c (Å) | 72.22, 68.85, 75.02 |
| α, β, γ (°) | 90, 110.27, 90 |
| Resolution (Å) | 48.29-2.50 (2.59-2.50) |
| $R_{merge}$ | 0.11 (0.60) |
| $I / \sigma I$ | 7.89 (2.46) |
| Redundancy | 3.4 (3.4) |
| Completeness (%) | 97.3 (92) |
| Wilson $B$-factor (Å$^2$) | 38.12 |
| | |
| **Refinement** | |
| Resolution (Å) | 48.29- 2.50 |
| No. reflections | 23447 |
| $R_{work}$ / $R_{free}$ | 0.21/0.26 |
| No. atoms | |
| Protein | 5007 |
| Ligand/ion | 2 |
| Water | 105 |
| $B$-factors | |
| Protein | 58.87 |
| Ligand/ion | 42.04 |
| Water | 41.57 |
| R.m.s. deviations | |
| Bond lengths (Å) | 0.007 |
| Bond angles (°) | 0.83 |
| Number of TLS groups | 26 |
| Ramachandran favored (%) | 96 |
| Ramachandran outliers (%) | 0 |
| Rotamer outlier (%) | 2.2 |
| Clashscore | 8.73 |
| PDB code | 6LHD |

## Supplementary Table 2. List of all primers

| |
|---|
| BCLxL clone to pET28a with C-terminal his tag |
| BCLXL-FW: TTTAAGAAGGAGATATACCATGTCTCAGAGCAACCGGGAG<br>BCLXL-REV: GGTGGTGGTGGTGATCGAGGCGTTCCTGGCCCTTTC |
| BCL2 clone to pET28a with C-terminal his tag |
| 28aBCL2-FW: CTTTAAGAAGGAGATATACCATGGCGCACGCTGGGAGAAC<br>28aBCL2-REV: GTGGTGGTGGTGGTGATCGAGCCGCATGCTGGGGCCGTAC |
| BCLxL clone topGEx-6P1: |
| GSTbclxl-fw: TGTTCCAGGGGCCCCTGATGTCTCAGAGCAACCGGGAG<br>GSTbclxl-rev: ACGATGCGGCCGCTCGAGTCAGCGTTCCTGGCCCTTTC |
| BCLxL clone to pQCXIH: |
| FW: GCAGGAATTGATCCGCGGCCGCATGTCTCAGAGCAACCGGGAG<br>REV: AGGGGCGGAATTCCGGATCCTCATTTCCGACTGAAGAGTG |
| Fused BCLxL-p53 clone to pET-28a: |
| XL53-FW1: GTTCTGTTTCAAGGCCCG AGCCAGAGCAATCGCGAAC<br>XL53-REV4: GTGGTGGTGCTCGAGTTATTTTCTGCGGAGATTCTCTTC<br>LINKER-2: CCAGAACCACGCGGAACCAGAGAACCACCTCCACCGGCTGCGGCATTGTTGCCG<br>LINKER-3: CTCTGGTTCCGCGTGGTTCTGGAGGTGGTGGTTCTTCTGTCCCTTCCCAGAAAAC |
| p53 Clone to pQCXIH: |
| p53FL-fw: TGCAGGAATTGATCCGCGGCCGCATGGAGGAGCCGCAGTCAG<br>p53FL-rev: AGAGGGGCGGAATTCCGGATCCTCAGTCTGAGTCAGGCCCTTC |
| p53-DT Clone to pET28a |
| P53-DT-FW: GTTCTGTTTCAAGGCCCGCCCCTGTCATCTTCTGTC<br>P53-DT-REV: GTGGTGGTGCTCGAGTTACCCTGGCTCCTTCCCAG |
| Mutations: |
| p53-Y107A-FW: CGCCGGTTTCCGTCTGGGCTTCTTGC<br>p53-Y107A-REV: CTGCCCTGGTAGGTTTTCTGGGAAG |
| p53-H178A-FW: CCGCACATGAGCGCTGCTCAGATAGCGATG<br>p53-H178A-REV: GGCAGCGCCTCACAACCTCCGTCATG |
| p53-R273A-FW: TGGCTGTTTGTGCCTGTCCTGGGAGAGACC<br>p53-R273A-REV: CCTCAAAGCTGTTCCGTCCCAGTAG |
| p53-R273C-FW: GACGGAACAGCTTTGAGGTGTGTGTTTGTGCCTGTCCTGGGAG<br>p53-R273C-REV: CTCCCAGGACAGGCACAAACACACACCTCAAAGCTGTTCCGTC |
| p53-R248A-FW: AACgcGAGGCCCATCCTCACCATCATCACAC<br>p53-R248A-REV: CATGCCGCCCATGCAGGAACTGTTAC |
| BCLxL-Y22A-FW: CTACAAGCTTTCCCAGAAAGGAGCCAGCTGGAGTCAGTTTAGTGATG<br>BCLxL-Y22A-REV: CATCACTAAACTGACTCCAGCTGGCTCCTTTCTGGGAAAGCTTGTAG |
| BCLxL-D156R-FW: GCACTGTGCGTGGAAAGCGTAAGAAAGGAGATGCAGGTATTGGTG<br>BCLxL-D156R-REV: CACCAATACCTGCATCTCCTTTCTTACGCTTTCCACGCACAGTGC |

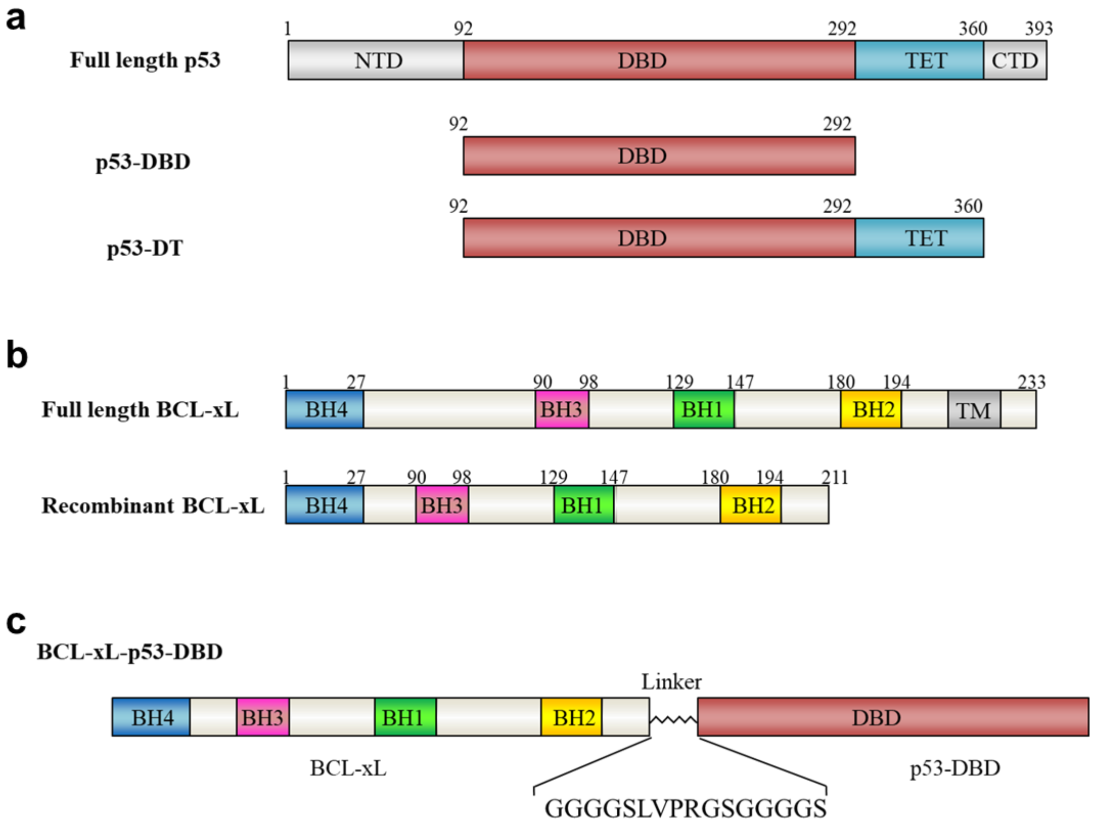


**Supplementary Fig. 1. Schematic representation of constructs. a** p53 constructs. **b** BCL-xL constructs. **c** Construct of fusion protein used in this study.

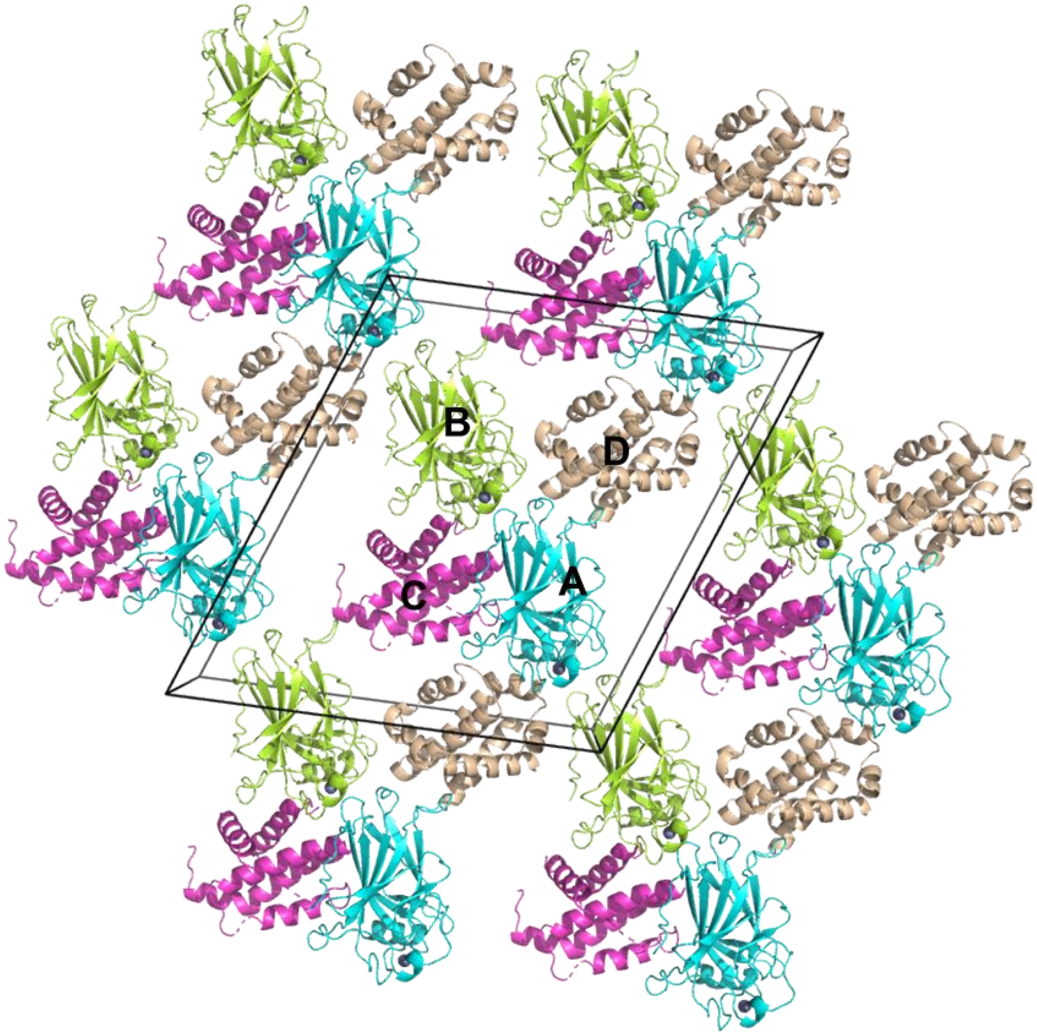


**Supplementary Fig. 2. Crystal packing in the p53/BCL-xL structure.**

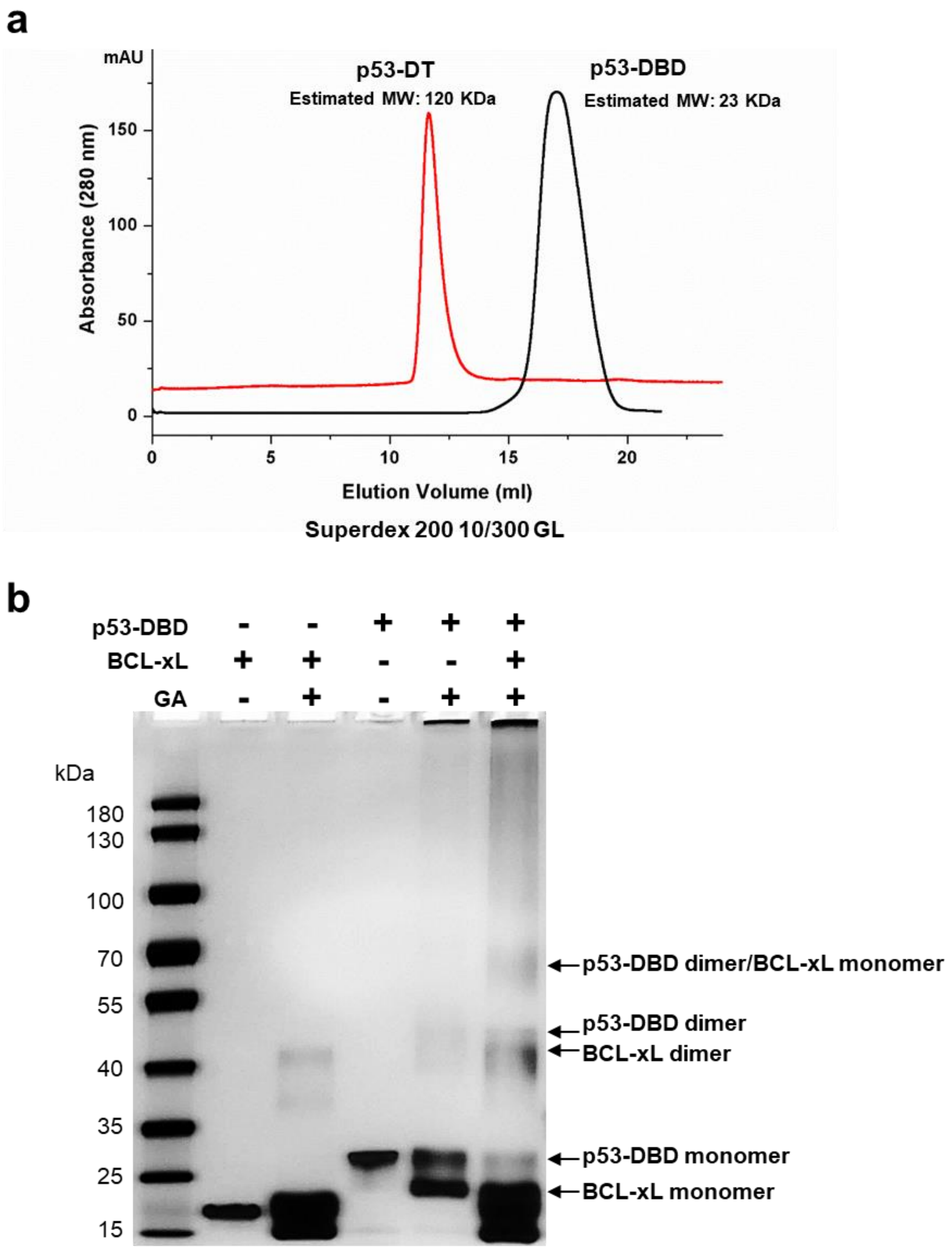


**Supplementary Fig. 3. Crosslinking assay of p53 binding to BCL-xL. a** Gel-filtration chromatography of p53 proteins. p53-DT (red line) was eluted at about 12 ml on column Superdex 200 10/300 GL, while p53-DBD (black line) was eluted at about 17 ml. The estimated molecular weight (MW) was shown as indicated. **b** The complex formation of p53-DBD and BCL-xL was analyzed by protein crosslinking and SDS-PAGE. 10 μM purified p53-DBD was incubated with 0.1% glutaraldehyde (GA) at 37°C for 15 min in the presence or absence of BCL-xL as indicated. Products of the crosslinking reactions were analyzed by SDS-PAGE followed by silver staining. The results of three independent replicates were similar, with one representative experiment being shown.

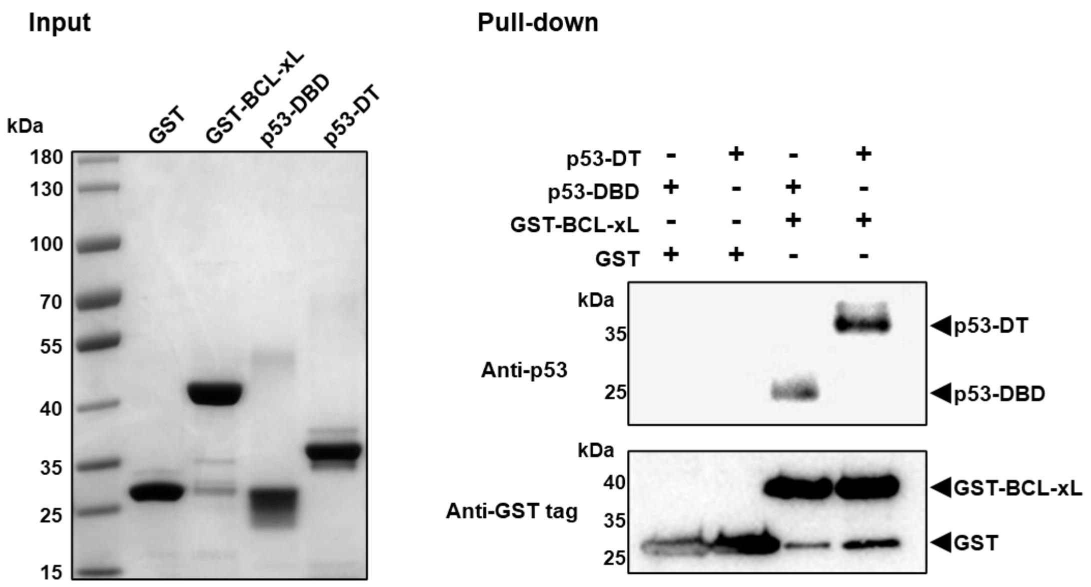


**Supplementary Fig. 4. GST pull-down showing p53 binding to BCL-xL.** Purified proteins were identified by SDS-PAGE and Coomassie blue staining (left). GST tag or GST-BCL-xL was incubated with p53-DBD or p53-DT protein as indicated (right). The mixture was incubated with GST beads, and detected by western blotting using anti-p53 antibody or anti-GST antibody. The results of three independent replicates were similar, with one representative experiment being shown.

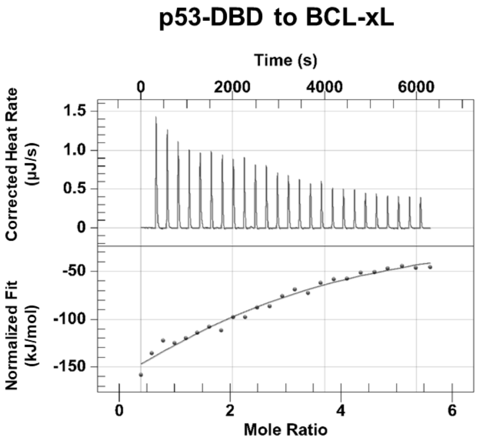


Kd (µM): 37.4±15.4
n: 2.2
△H (kJ/mol): -370±35
T△S (kJ/mol): -348±37

**Supplementary Fig. 5. Representative isothermal titration calorimetry (ITC) thermographs for titration of p53-DBD proteins to BCL-xL.** The integrated thermodynamic parameters are list below shown as average of three independent titrations ± SEM.

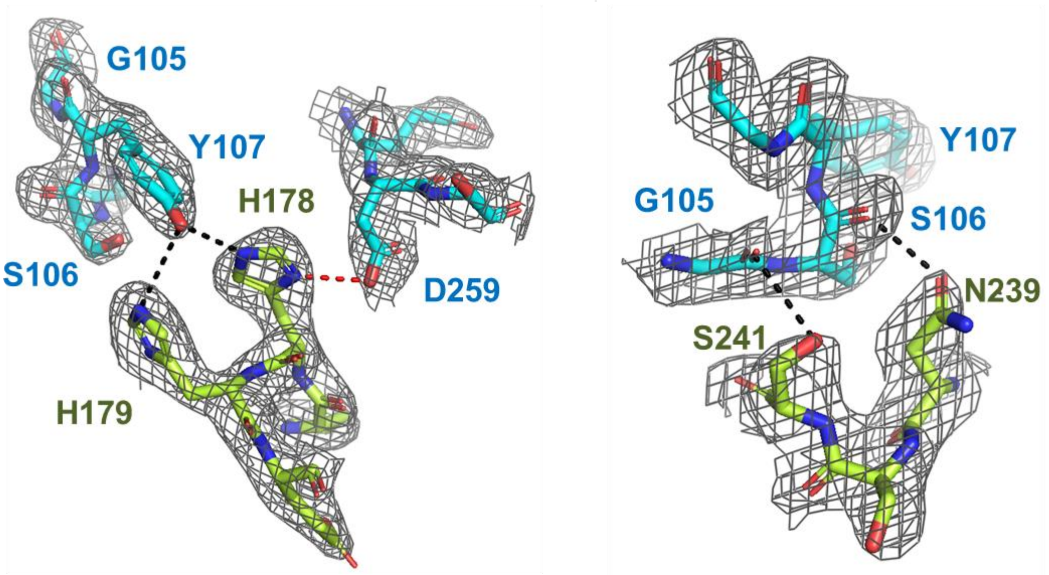


**Supplementary Fig. 6. Electron density map of the p53-dimer interface.** An electron density map (2mFo-DFc) at 1σ shows the key intermolecular hydrogen bonds (black dashed line) and salt bridges (red dashed line) in the p53 dimer interface**.** p53-A: limon; p53-B: cyan.

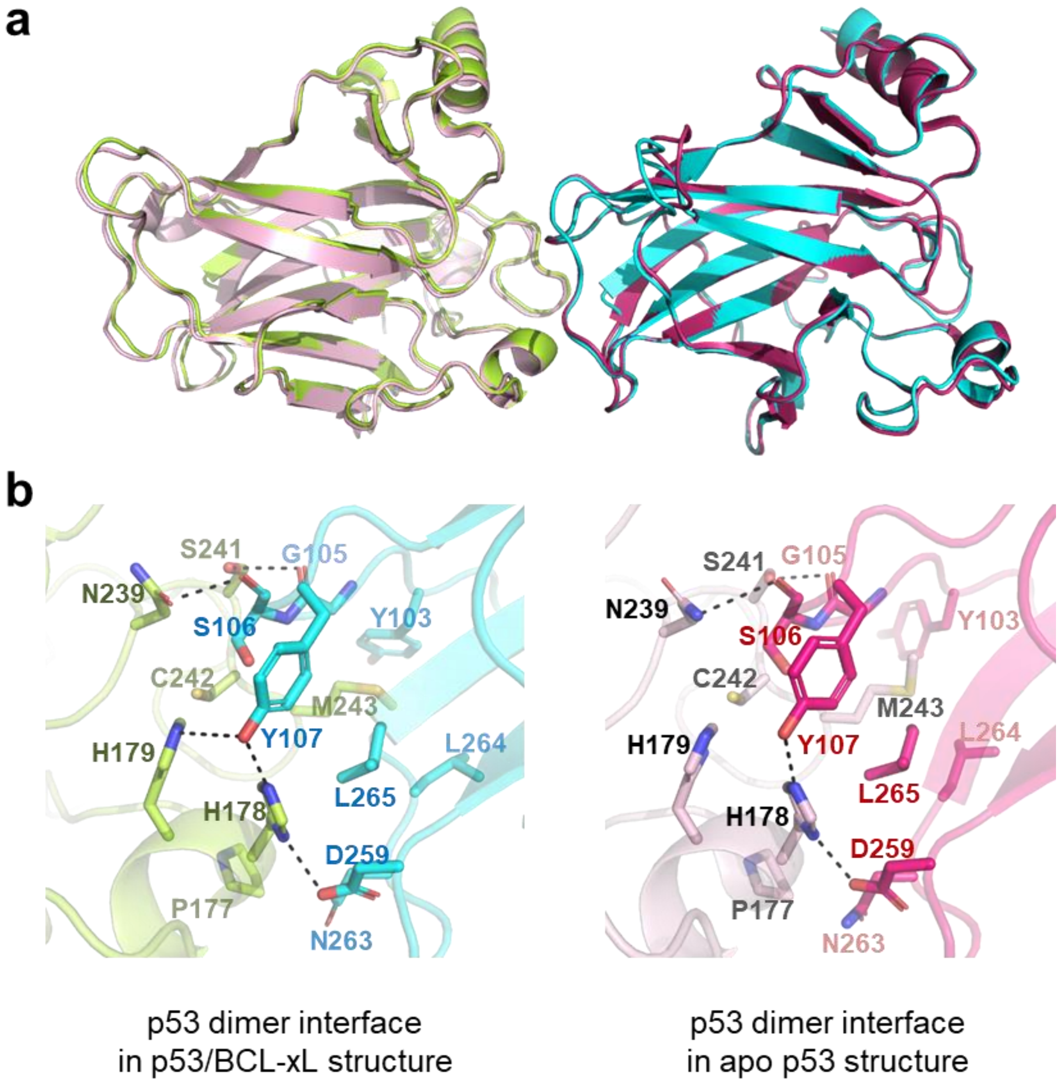


**Supplementary Fig. 7**. **Comparison of p53 dimer interfaces in the p53/BCL-xL complex and in the apo p53-DBD structure (PDB: 2OCJ). a**. Superposition of A-B molecules (A: limon, B: cyan) in the p53/BCL-xL complex with B-D molecules (B: lightpink, D: hotpink) in the apo p53-DBD structure. **b**. Detailed interfacial residues in the p53 dimer interfaces.

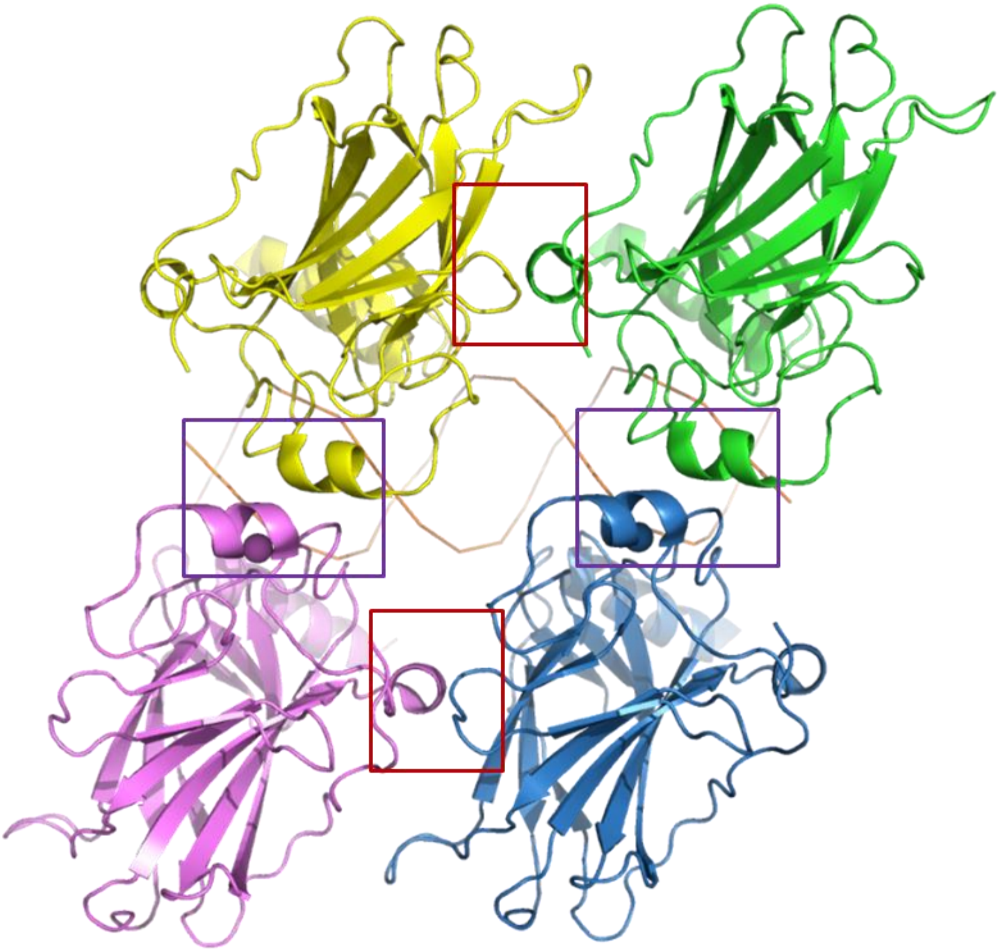

p53-DBD/DNA complex

**Supplementary Fig. 8. p53-DBD dimer interface and dimer-dimer interface in the p53-DBD/DNA complex (PDB: 3KMD).** Purple boxes indicate the dimer interface; red boxes indicate the dimer-dimer interface.

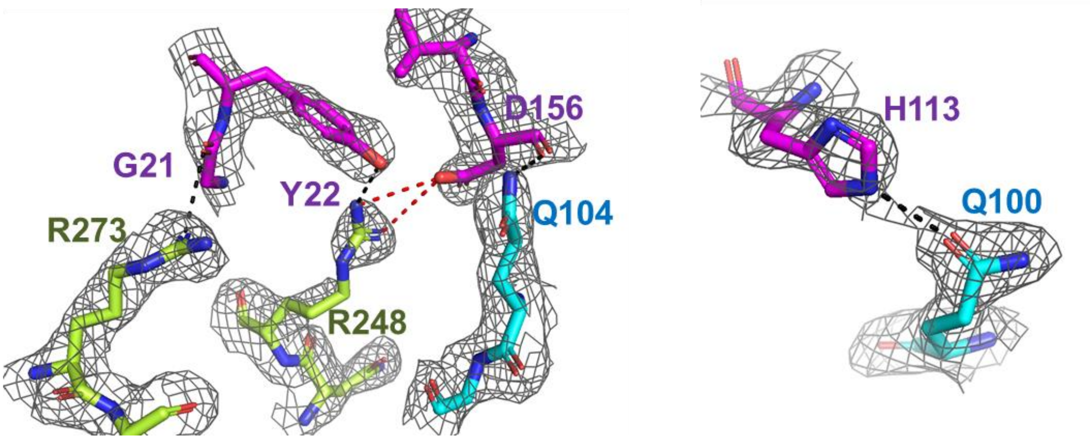


**Supplementary Fig. 9. Electron density map of the BCL-xL/p53 binding interface.** An electron density map (2mFo-DFc) at 1σ shows the key intermolecular hydrogen bonds (black dashed line) and salt bridges (red dashed line) in the BCL-xL binding interface**.** p53-A: limon; p53-B: cyan; BCL-xL: magenta.

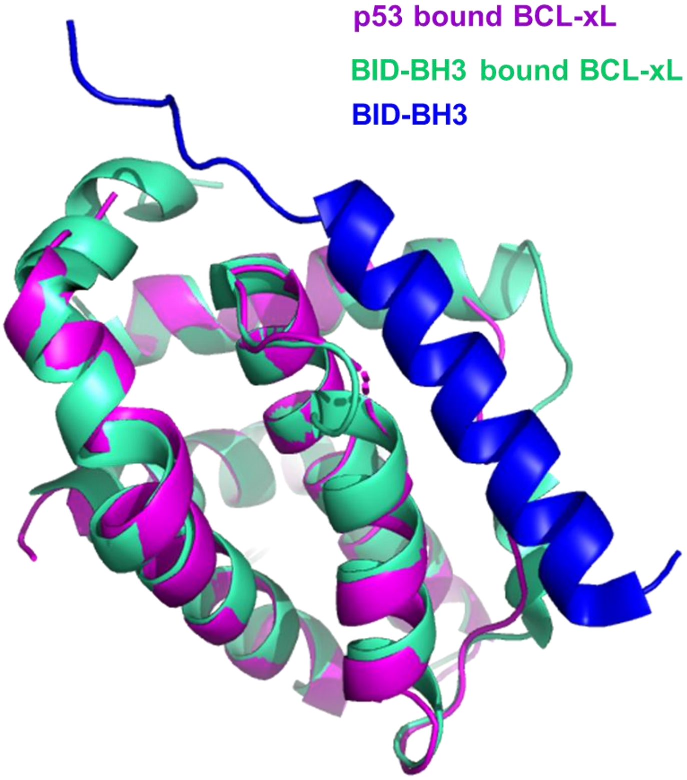


**Supplementary Fig. 10. Comparison of p53-bound BCL-xL (magenta) in the p53/BCL-xL structure with BID-BH3 bound BCL-xL (PDB: 4QVE, limegreen).** The RMSD for the superposition of the BCL-xL molecules is 0.66Å.

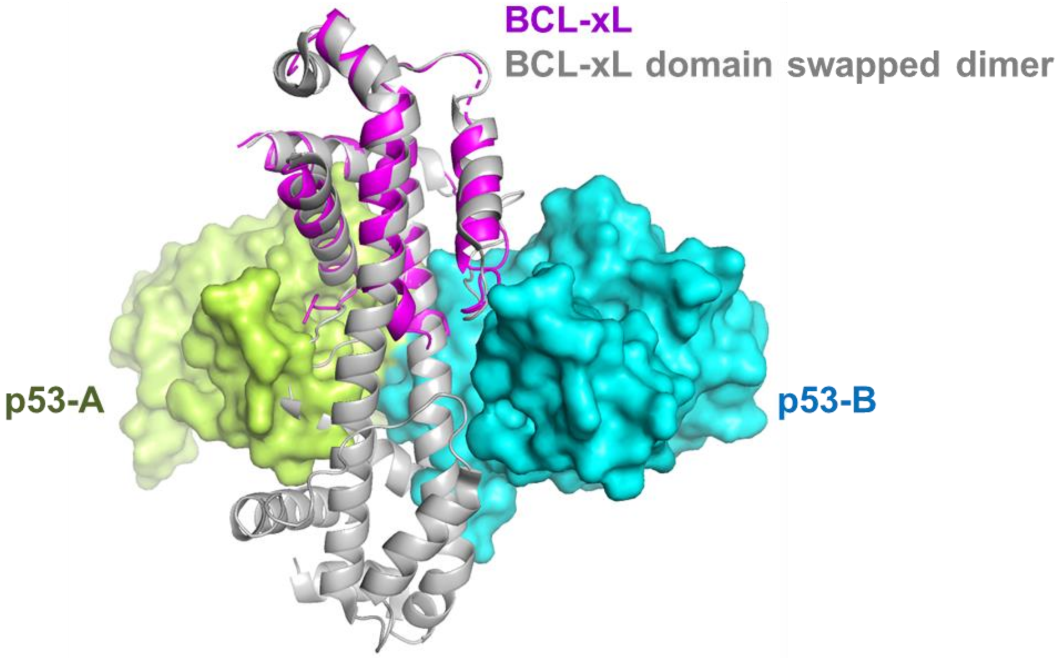


**Supplementary Fig. 11. Superposition of BCL-xL (magenta) in the p53/BCL-xL complex with a 3D-domain swapped dimer of BCL-xL homodimer (PDB: 4PPI, gray).**

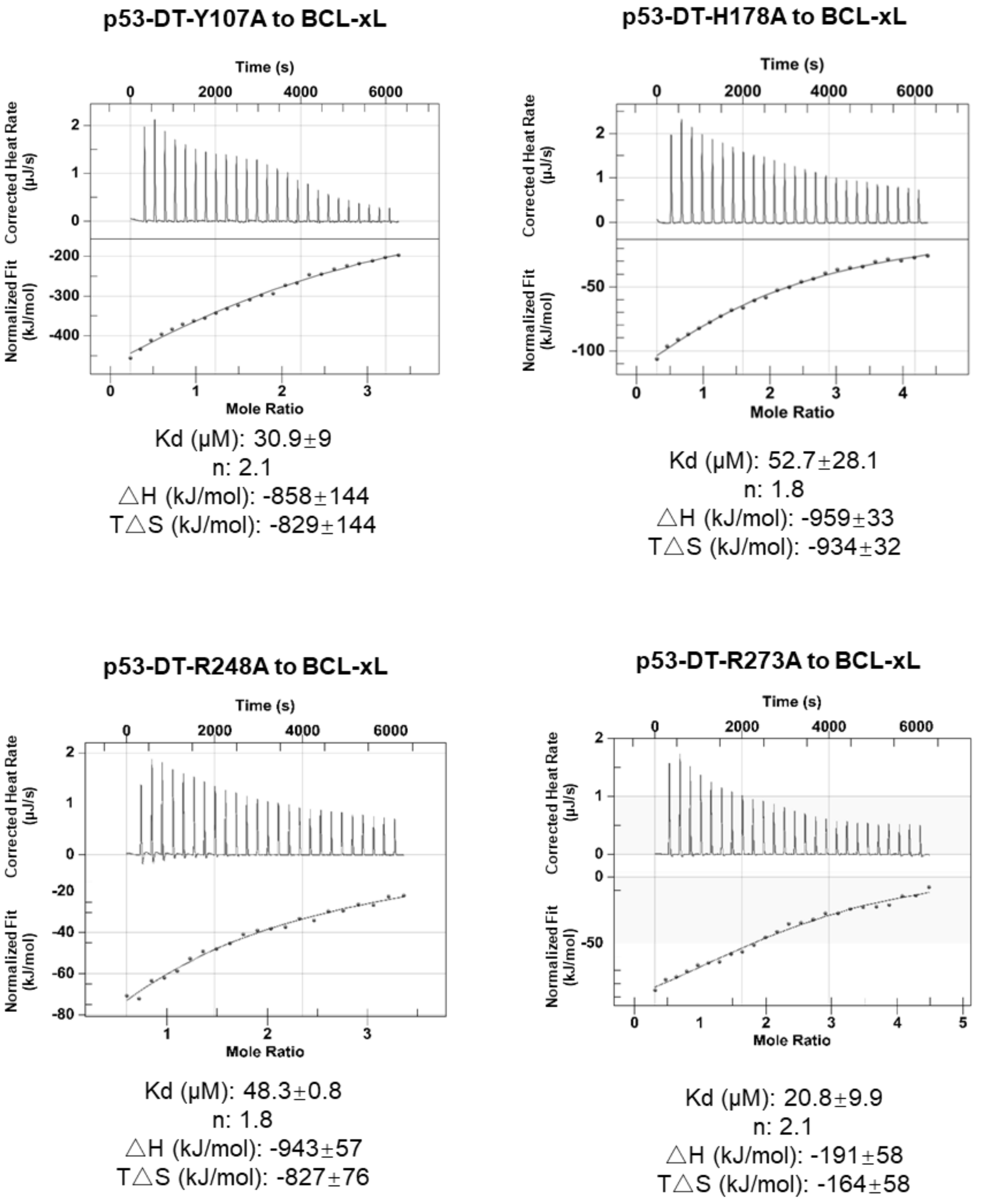


**Supplementary Fig. 12. Representative isothermal titration calorimetry (ITC) thermographs for titration of p5-DT mutants to BCL-xL.** The integrated thermodynamic parameters are list below shown as average of three independent titrations ± SEM.

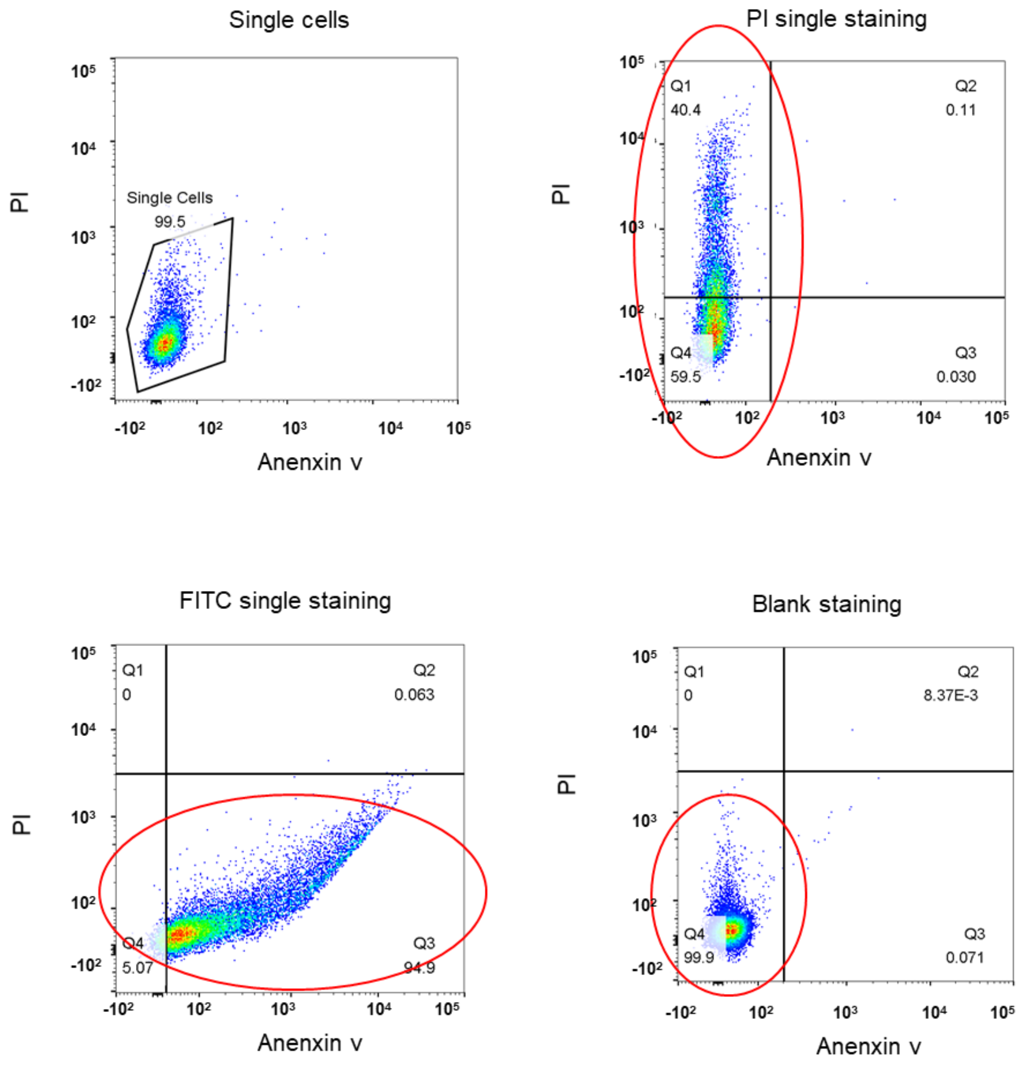


**Supplementary Fig. 13. Gating strategy for sorting apoptosis cells**. For the assessment of apoptosis, H1299 cells were trypsinized, washed with 1× phosphate-buffered saline (PBS) and stained using the FITC Annexin-V Apoptosis Detection Kit with PI (Vazyme, A211-01), following the manufacturer's instructions. Stained cells and controls (FITC Annexin-V only, PI only and blank) were then processed by fluorescence-activated cell sorting (Cytek Dxp Athena flow cytometer) and analyzed by FlowJo software. Each experiment was repeated three times with similar results, with one representative experiment being shown.

**p53-DT to BCL-2**

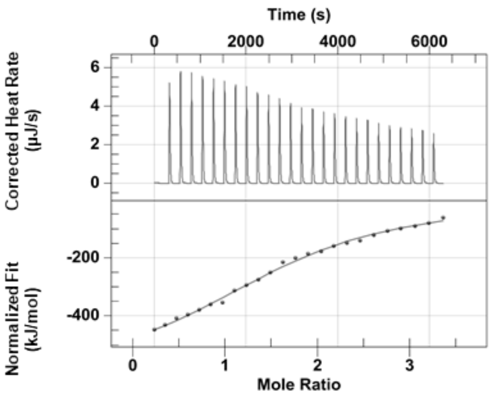


Kd (μM):3.3±0.8
n: 1.7
△H (kJ/mol): -459±79
T△S (kJ/mol): -428±80

**Supplementary Fig. 14. Representative isothermal titration calorimetry (ITC) thermographs for titration of p5-DT to BCL-2.** The integrated thermodynamic parameters are list below shown as average of three independent titrations ± SEM.

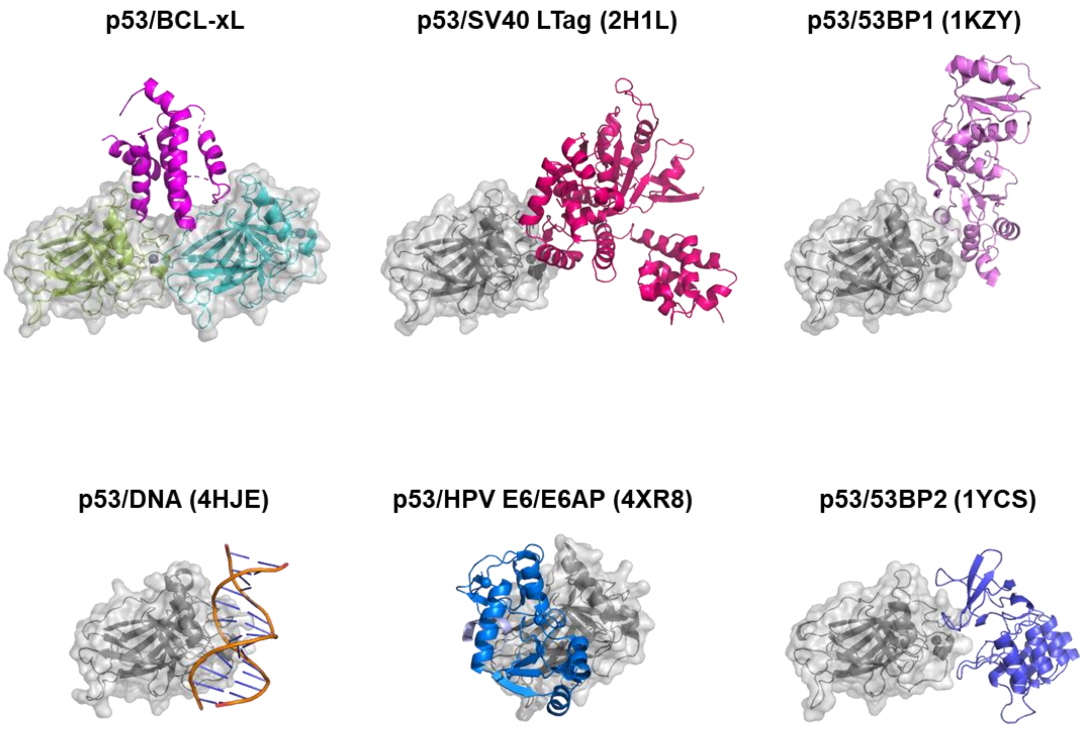


**Supplementary Fig. 15**. **Comparison of the structure of p53/BCL-xL complex with other structures of p53 complex.** Surface models of p53 are colored gray. These binding partners of p53 are shown as cartoon. DNA: orange; SV40 LTag: warm pink; 53BP1: violet; 53BP2: blue; HPV E6: marine; E6AP: light blue. The PDB code of structures are presented in bracket.
